# Dissecting miRNAs in Wheat D Genome Progenitor, *Aegilops tauschii*

**DOI:** 10.3389/fpls.2016.00606

**Published:** 2016-05-04

**Authors:** Bala A. Akpinar, Hikmet Budak

**Affiliations:** ^1^Molecular Biology, Genetics and Bioengineering Program, Faculty of Engineering and Natural Sciences, Sabanci UniversityIstanbul, Turkey; ^2^Department of Plant Sciences and Plant Pathology, Montana State UniversityBozeman, MT, USA

**Keywords:** *Aegilops tauschii*, *Triticum aestivum*, microRNA, D genome, drought, next generation sequencing

## Abstract

As the post-transcriptional regulators of gene expression, microRNAs or miRNAs comprise an integral part of understanding how genomes function. Although miRNAs have been a major focus of recent efforts, miRNA research is still in its infancy in most plant species. *Aegilops tauschii*, the D genome progenitor of bread wheat, is a wild diploid grass exhibiting remarkable population diversity. Due to the direct ancestry and the diverse gene pool, *A. tauschii* is a promising source for bread wheat improvement. In this study, a total of 87 *Aegilops* miRNA families, including 51 previously unknown, were computationally identified both at the subgenomic level, using flow-sorted *A. tauschii* 5D chromosome, and at the whole genome level. Predictions at the genomic and subgenomic levels suggested *A. tauschii* 5D chromosome as rich in pre-miRNAs that are highly associated with Class II DNA transposons. In order to gain insights into miRNA evolution, putative 5D chromosome miRNAs were compared to its modern ortholog, *Triticum aestivum* 5D chromosome, revealing that 48 of the 58 *A. tauschii* 5D miRNAs were conserved in orthologous *T. aestivum* 5D chromosome. The expression profiles of selected miRNAs (miR167, miR5205, miR5175, miR5523) provided the first experimental evidence for miR5175, miR5205 and miR5523, and revealed differential expressional changes in response to drought in different genetic backgrounds for miR167 and miR5175. Interestingly, while miR5523 coding regions were present and expressed as pre-miR5523 in both *T. aestivum* and *A. tauschii*, the expression of mature miR5523 was observed only in *A. tauschii* under normal conditions, pointing out to an interference at the downstream processing of pre-miR5523 in *T. aestivum*. Overall, this study expands our knowledge on the miRNA catalog of *A. tauschii*, locating a subset specifically to the 5D chromosome, with ample functional and comparative insight which should contribute to and complement efforts to develop drought tolerant wheat varieties.

## Introduction

*Aegilops tauschii* (goat grass) is the D genome progenitor of hexaploid bread wheat. About 8,000 years ago, its spontaneous hybridization with the cultivated allotetraploid *Triticum turgidum* in the Fertile Crescent resulted in an allohexaploid, currently known as *Triticum aestivum* (bread wheat; Brenchley et al., 2012). Bread wheat, being the major staple food in the world, occupies 17% of all the cultivated land and meets nearly 20% of the human dietary energy supply (Lucas and Budak, 2012). Since biotic and abiotic stresses such as drought, are limiting factors to wheat yield and quality, much effort has been put on elucidating the molecular background of stress responses (Ergen and Budak, 2009; Xin et al., 2010; Lucas et al., 2011, 2012; Brenchley et al., 2012; Kuzuoglu-Ozturk et al., 2012; Budak et al., 2014). The allohexaploid nature of its genome challenges genetics and genomics research on bread wheat. Fortunately, the genome sequencing of its A and D genome progenitors, *Triticum urartu* and *A. tauschii*, has delivered important insight into wheat genome structure, organization and evolution, and provided a valuable resource for the wheat community, both for further genomics research and improvement (Jia et al., 2013; Ling et al., 2013).

MicroRNAs, or miRNAs, are small, non-coding RNAs that aid in post-transcriptional gene regulation with essential roles in key biological pathways (Budak and Akpinar, 2015). They regulate their own biogenesis and are involved in various processes such as development, differentiation, response to stress, genome maintenance, and integrity (Mallory and Vaucheret, 2006; Wilusz et al., 2009; Lucas and Budak, 2012; Kurtoglu et al., 2013; Akpinar et al., 2015a; Budak et al., 2015c). It has been a decade since the discovery of the first plant miRNA by Llave et al. (2002). By this time, identification and characterization of several small RNAs including miRNAs from various species have unlocked the miRNA contents of plants, thereby improving our understanding of the regulation of key biological processes (Kawaji and Hayashizaki, 2008; Yao and Sun, 2012; Budak et al., 2014, 2015a; Budak and Akpinar, 2015; Alptekin and Budak, 2016). In terms of wheat species, most research groups have initially focused on the identification of bread wheat miRNAs, and the miRNA catalogs of wild wheat species or wheat relatives have just begun to be explored. In general, miRNA identification studies follow either one or a combination of two main strategies: experimental and computational identification (Yao et al., 2007; Kantar et al., 2011b). Experimental approach adopting sequencing of small RNA libraries resulted in the identification of several wheat miRNAs, including *Aegilops* miRNAs (Yao et al., 2007; Wei et al., 2009; Xin et al., 2010; Kenan-Eichler et al., 2011; Gupta et al., 2012; Tang et al., 2012; Jia et al., 2013; Li et al., 2013). Additionally, computationally identified *Aegilops* miRNAs were also reported from a relatively limited pool of genomic sequences (Dryanova et al., 2008). In contrast, several advanced *in silico* miRNA identification studies were undertaken for *T. aestivum* (Dryanova et al., 2008; Yin and Shen, 2010; Schreiber et al., 2011; Pandey et al., 2013), including those that has been performed at the subgenomic level, focusing on 1AL, 4A, 5A, and 5D chromosomes (Vitulo et al., 2011; Kantar et al., 2012; Lucas and Budak, 2012; Kurtoglu et al., 2013).

Several of the above mentioned *in silico* methods have utilized the NGS data, accumulated by the latest breakthrough in sequencing technologies (Vitulo et al., 2011; Hernandez et al., 2012; Kantar et al., 2012; Lucas and Budak, 2012; Kurtoglu et al., 2014; Akpinar et al., 2015b). miRNA identification at the subgenomic level has also taken advantage of the recently developed chromosome flow sorting technique, which reduces the complex and repetitive genomes to a manageable size (Vrána et al., 2000, 2012; Kubaláková et al., 2002). These innovations enabled major progresses in understanding plant genomes, and speeding up miRNA identification studies.

In this study, homology-based *in silico* method was adopted for the identification of *A. tauschii* miRNAs at both genomic and subgenomic levels. For a comprehensive miRNA analysis, flow sorted 5D chromosome reads, recently sequenced by our group and whole genome assembly data were used (Jia et al., 2013; Akpinar et al., 2014). In order to gain insights into subgenomic miRNA evolution, we also compared *Aegilops* 5D miRNAs with bread wheat 5D miRNAs previously published by our group (Kurtoglu et al., 2013; Lucas et al., 2014). Finally, experimental verification and quantification of selected miRNAs in response to drought were also performed with qRT-PCR.

## Materials and Methods

### Plant miRNA Reference Set and Genomic Sequences

Mature miRNA sequences of 67 different *Viridiplantae* species were downloaded from miRBase release 20 (June 2013; Kozomara and Griffiths-Jones, 2011). Of multiple miRNAs having the same mature miRNA sequence only one was retained. The resulting dataset containing 3,228 unique mature miRNA sequences was used as query in *A. tauschii* miRNA prediction.

The whole genome assembly of *A. tauschii* was constructed by SOAP *de novo* from the genomic Illumina reads of accession AL8/78 (Jia et al., 2013) and is publicly available at: http://www.ebi.ac.uk/ena/data/view/AOCO01000000. *A. tauschii* 5D chromosome was previously purified and sequenced by our group (Akpinar et al., 2014). Briefly, a shotgun library was produced from 0.5 μg flow sorted chromosome and sequenced using GS FLX Titanium kits according to the manufacturer’s protocols (all Roche 454 Life Sciences). The whole genome assembly contained 7,107,581 contigs, and the 5D chromosome data was comprised of 1,477,789 reads representing 0.8x coverage of the chromosome.

### *In Silico* miRNA Identification Based on Sequence Similarity and Secondary Structure Conservation

For *in silico* miRNA identification, we adopted a homology-based method with a two-step procedure: preliminary selection of *A. tauschii* sequences with homology to a previously known plant mature miRNA and subsequent elimination based on the consistency of candidate stem–loop secondary structures in relation to the general, pre-established precursor miRNA (pre-miRNA) features (Ambros et al., 2003; Unver and Budak, 2009; Kantar et al., 2010, 2011b, 2012; Lucas and Budak, 2012). Two in-house Perl scripts, SUmirFind and SUmirFold, used for the homology-based prediction of putative miRNAs were described in detail in our previous publications (Unver and Budak, 2009; Kantar et al., 2010, 2012; Lucas and Budak, 2012; Kurtoglu et al., 2013). Briefly, BLAST+ stand-alone toolkit, version 2.2.25 (March 2011) was used to generate databases for two *Aegilops* sequence datasets (Camacho et al., 2009). The plant miRNA reference set was searched against these *A. tauschii* databases using SUmirFind script with a maximum of 3 allowed mismatches. Candidate sequences exhibiting significant similarity to known miRNA sequences were then evaluated by SUmirFold in terms of secondary structure features and lowest MFE (Markham and Zuker, 2008). As the first step, SUmirFold eliminates candidate sequences based on a mismatch cutoff for the miRNA:miRNA^∗^ duplex: 4 for miRNA and 6 for miRNA^∗^. For all sequences passing this step, the program excises and re-folds the regions around the duplexes and evaluates the foldback structures against pre-established pre-miRNA characteristics (Ambros et al., 2003; Unver and Budak, 2009; Kantar et al., 2010, 2011b, 2012; Lucas and Budak, 2012). The candidates passing SUmirFold were further inspected based on the following criteria: Hairpins cannot have (1) multi-branched loops, or (2) inappropriate DICER cut sites at the ends of the miRNA-miRNA^∗^ duplex; (3) mature miRNA sequence cannot be located at the head section of the hairpin; (4) large loops failing the miRNA-miRNA^∗^ duplex mismatch criteria but were skipped by SUmirFold due to unclear miRNA^∗^ start or end sites are not allowed. As a final step, redundant hits, resulting from identical miRNAs were predicted from two similar query mature miRNA sequences were also excluded from the final dataset. All mature miRNA and pre-miRNA sequences of the newly predicted miRNAs are given in Supplementary Table S1.

### Repeat Analysis of Putative Pre-miRNAs

Repetitive elements were identified by a semi-automated pipeline, RepeatMasker version 3.2.9^1^ at default settings with Cross-Match^2^ as an alignment algorithm. MIPS-REdatPoaceae v9.3p^3^ repeat element database containing 34,135 sequences was used as the repeat library. Pre-miRNAs covered more than 50% or their lengths by repetitive elements were considered as ‘repeat-related,’ while the remaining were denoted as ‘nonrepeat-related.’ miRNAs which have both ‘repeat-related’ and ‘nonrepeat-related’ stem–loops were termed as ‘low confidence,’ and others only corresponding to hairpins with non-repetitive content were termed as ‘high confidence.’

### Genomic Representation Analysis of Putative Pre-miRNAs

Genomic representation (referred as ‘representation’ hereafter) analysis was performed independently for three different miRNA datasets: *A. tauschii* whole genome assembly and *A. tauschii* 5D chromosome miRNAs, identified in this study, and *T. aestivum* 5D chromosome miRNAs, retrieved from a recent publication of our group (Kurtoglu et al., 2013). The number of ‘repeat-related’ and ‘nonrepeat-related’ hairpins corresponding to each miRNA was counted and their representations were separately recorded. miRNA representation was calculated as the total number of hairpins from different genomic locations. Pre-miRNAs that were identical in sequence were also included in the overall representation if they were found to originate from different sequences, or in different positions of the same assembly sequence. Additionally, identical pre-miRNA sequences located on the same genomic position, differ in terms of their mature miRNA locations were also retained.

The percent representations of different miRNAs in the overall hairpin pool of each dataset were calculated. For each miRNA, its comparative representation across datasets was also assessed. This analysis was based on the assumption that total miRNA pools of datasets were in proportion with the length of the chromosome/genome (*Aegilops* whole genome: 4.03 Gb; *Aegilops* 5D: 577 Mb; *T. aestivum* 5D: 748 Mb) targeted in each dataset (Safár et al., 2010; Luo et al., 2013). In order to compare the representations of common 5D miRNAs across two species, whole repertoire of 5D hairpins from *Aegilops* and wheat were accepted as 577 and 748 units, respectively. The representation of each miRNA was expressed as units. Representations of common miRNAs between the *Aegilops* 5D and *Aegilops* whole genome assembly datasets were also compared. In this analysis, overall representation of 5D miRNAs was assumed to constitute 14.32% of the whole miRNA pool of *Aegilops.*

### Expression and Target Analysis of *Aegilops* miRNAs

*In silico* expression analysis of *Aegilops* whole genome and 5D miRNAs was performed by searching the predicted hairpins against two different expressed sequence databases: (1) *Aegilops* transcriptome assembly retrieved from Jia et al. (2013); (2) *A. tauschii* ESTs retrieved from NCBI(taxid: 37682). Similarity searches were performed on BLAST+ stand-alone toolkit, version 2.2.25+ release (March 2011; Camacho et al., 2009) for the *Aegilops* transcriptome assembly, and on NCBI BLASTN megablast web-tool, in settings optimized for the detection of highly similar sequences for ESTs. The results of both searches were combined and further filtered for 98% identity and 99% query coverage.

The expressed sequence databases used in the expression analysis of putative miRNAs were also utilized in the target prediction. Targets for the 51 *Aegilops* miRNAs reported in this study were predicted using psRNATarget web-tool^4^ (Dai and Zhao, 2011; Kantar et al., 2012). The corresponding proteins were identified by similarity searches against *A. tauschii* (taxid: 37682) non-redundant protein database using NCBI BLASTX tool (98% similarity and 99% query coverage). Finally, QuickGO^5^, a web-browser for gene ontology terms and annotations, was used to assign functions to the protein putatively targeted by miRNAs.

### Plant Materials, Growth Conditions, and Application of Dehydration Stress

*A. tauschii* and *T. aestivum* var. CS seeds were vernalized for 4 days at 4°C. Seedlings were then sown to soil supplemented with 200 ppm N, 100 ppm P, and 20 ppm S and grown in conditions previously described by Kurtoglu et al. (2013). Shock dehydration stress treatment was applied to three sets of seedlings: one leaf stage wheat (dap: 7), two leaf stage wheat (dap: 17) and two leaf stage *Aegilops* (dap: 7). Stress application was performed by leaving the plants on paper towels for 4 h (Ergen et al., 2009). Whole seedlings of control and stressed plants were collected and their tissues were fast frozen in liquid nitrogen, and stored at -80°C (Kantar et al., 2010).

### Verification and Quantification of Selected miRNAs via qRT-PCR

Total RNA was isolated from stressed and control whole seedlings of *A. tauschii* and *T. aestivum* using TRI Reagent (Sigma, St. Louis, MO, USA) following the manufacturer’s instructions. RNA integrity was verified by separating the major ribosomal RNA bands in 2% agarose gels. To eliminate contaminating gDNA, 1 μg of total RNA samples were treated with 1 U of DNase I (Fermentas) in a 10 μl reaction mix, and incubated at 37°C for 30 min. The reaction was terminated by adding 1 μl of 25 mM EDTA, followed by incubation at 85°C for 10 min.

Genomic DNA from *A. tauschii* and *T. aestivum* samples was isolated using Wizard^®^ Genomic DNA Purification Kit (Madison, WI, USA), according to manufacturer’s recommendations. All nucleic acid samples were quantified using Nanodrop ND-100 spectrophotometer (Nanodrop Technologies, Wilmington, DE, USA) and stored at -20°C.

First strand cDNA synthesis was performed on 100 ng of DNase treated RNA samples using RevertAid H Minus Reverse Transcriptase (EP0451; Fermentas) according to manufacturer’s protocols. Stem–loop RT primers for miR167, miR5175, miR5205, and miR5523 were designed according to Varkonyi-Gasic et al. (2007; Supplementary Table S2). miRNA-specific stem–loop reverse transcription reactions were performed using RevertAid H Minus Reverse Transcriptase (EP0451; Fermentas). The reaction mix containing 1 μl of DNase treated RNA (100 ng), 1 μl of 1 μM stem–loop RT primer (final concentration: 50 nM) and 9 μl DEPC-treated water was incubated at 70°C for 5 min, and immediately chilled on ice. Afterward, 4 μl reaction buffer (5×), 2 μl 10 mM dNTP mix (final concentration: 1 mM), 0.5 μl Ribonuclease Inhibitor (20 U) were added to the reaction mix and the final volume was completed to 19 μl with DEPC-treated water. This mix was incubated at 37°C for 5 min. After the addition of 1 μl of RevertAid H Minus M-MuLV Reverse Transcriptase (200 U), 20 μl RT reaction was performed using the following conditions: 30 min at 16°C, 60 cycles at 30°C for 30 s, 42°C for 30 s, and 50°C for 1 s. The reactions were terminated at 70°C for 10 min. As negative controls, no-RT primer and no-RNA control reactions were also included.

In order to experimentally verify selected miRNAs, miR167, miR5175, miR5205 and miR5523, and quantify their expression levels in response to 4 h shock drought stress qRT-PCR using FastStart Universal SYBR Green Master (ROX; Mannheim, Germany) was performed with the following reaction mixture: 20 μl reaction included 3 μl RT stem–loop cDNA products, 10 μl 2× Master mix, 0.6 μl primers (300 nM each) and 6.4 μl nuclease-free water. miRNA specific forward primers were designed for each miRNA and a universal reverse primer (5′-GTGCAGGGTCCGAGGT-3′) was used (Varkonyi-Gasic et al., 2007; Supplementary Table S2). qRT-PCR reactions were performed in iCycler iQ^TM^ Real-Time PCR Detection Systems (Bio-Rad Laboratories). Thermal cycling conditions were as follows: heated to 95°C for 10 min, followed by 40 cycles of 95°C for 15 s, 56/58°C for 30 s, and 72°C for 30 s, followed by 72°C for 7 min. The annealing temperatures were optimized to 56°C for miR5523 and 58°C for miR167, miR5175, and miR5205. The melting curves were generated by continuously collecting fluorescence signals from 65 to 95°C as the temperature increased at 0.2°C per second. All reactions were performed as triplets; no-RT primer and no-RNA controls were included. LinRegPCR program was used for polymerase chain reaction (PCR) efficiency calculations and quantification (Ruijter et al., 2009).

### Conservation of miR5523 Coding Regions and Pre-miR5523 Expression

Coding regions for pre-miR5523 and pre-miRNA expression were further analyzed in both *A. tauschii* and *T. aestivum* with conventional PCR. Putative pre-miR5523 genomic regions were screened in gDNAs and flow sorted 5D chromosomes of *Aegilops* and *T. aestivum*. Additionally, in control and drought stressed whole seedlings of *T. aestivum* (one leaf stage and two leaf stage) and in *Aegilops* (two leaf stage), pre-miR5523 expression was checked.

Polymerase chain reaction amplifications were carried out in a 20 μl PCR mix containing 1 μl (10 ng/μl) DNA/cDNA template, 2 μl 10× Taq buffer (final concentration 1×), 2 μl 25 mM MgCl_2_ (final concentration: 2.5 mM), 1.6 μl 2.5 mM dNTP (final concentration 0.2 mM), 0.6 μl 10 μM primer mix (final concentration: 300 nM each) and 0.1 μl of 5 U/μl Taq polymerase (0.5 U). Amplification reactions were performed in thermal cycler using the following conditions: 95°C for 5 min; 35 cycles of 95°C for 1 min, 56°C for 30 s, and 72°C for 30 s; followed by 72°C for 10 min. Forward and reverse primers are given in Supplementary Table S2. PCR products (with 1:5 μl 6× loading dye) were separated at 100 V in 3% agarose gels.

## Results

### Putative miRNAs Encoded By *A. tauschii* 5D Chromosome

Homology-based *in silico* miRNA prediction from a total of 1,477,789 chromosome-specific sequence reads of *A. tauschii* 5D chromosome suggested the presence of 3,055 pre-miRNA sequences, of which 2,601 were unique, along the 5D chromosome, putatively coding for 58 different miRNA families (**Tables 1** and **2**). All mature and pre-miRNA sequences of predicted miRNAs are given in Supplementary Table S1.

**Table 1:** Overall statistics of miRNA prediction.

	Ata-5D^1^	CS-5D^2^	Ata-WGA^3^
Sequence reads^a^/contigs^b^	1,477,789^a^	3,208,630^a^	7,107,581^b^
No of SUmirFind Hits	19,662	30,151	21,973
No of SUmirFold Hits	4,366	6,471	6,902
No. of miRNAs	58	60	80
Overall representation	3,055	4,691	4,868
Representation of non-repetitive hairpins	129	309	993
Representation of repetitive hairpins	2,913	4,382	3,875
No. of high confidence miRNAs	9	8	26
No. of low confidence miRNAs	6	12	8
No. of other miRNAs	43	40	46

*^1^Ata-5D: Aegilops tauschii 5D chromosome, ^2^CS-5D: Chinese Spring 5D chromosome, ^3^Ata-WGA: A. tauschii whole genome assembly, ^a^454 reads, ^b^Contigs of the assembly generated from Illumina reads.*

**Table 2:** MicroRNA (miRNA) coding sequences predicted to be present in *Aegilops* 5D and Wheat 5D chromosomes.

miRNAs found only in *Aegilops* 5D	Conserved miRNAs between *Aegilops* and wheat 5D chromosomes				miRNAs found only in wheat 5D
miR156	miR1117	miR1136	miR5021	miR5203	miR1123
miR158	miR1118	miR1137	miR5049	miR5205	miR169
miR319	miR1120	miR1139	miR5067	miR5281	miR2275
miR5057	miR1121	miR1436	miR5070	miR5387	miR3700
miR5069	miR1122	miR1439	miR5085	miR5568	miR395
miR5183	miR1125	miR160	miR5086	miR6191	miR398
miR5293	miR1127	miR166	miR5161	miR6197	miR5068
miR6205	miR1128	miR167	miR5169	miR6219	miR7775
miR6233	miR1130	miR1847	miR5174	miR6220	miR8036
miR6248	miR1131	miR2118	miR5175	miR6224	miR834
	miR1133	miR437	miR5180	miR7714	miR845
	miR1135	miR482	miR5181	miR818	miR950

**FIGURE 1:**
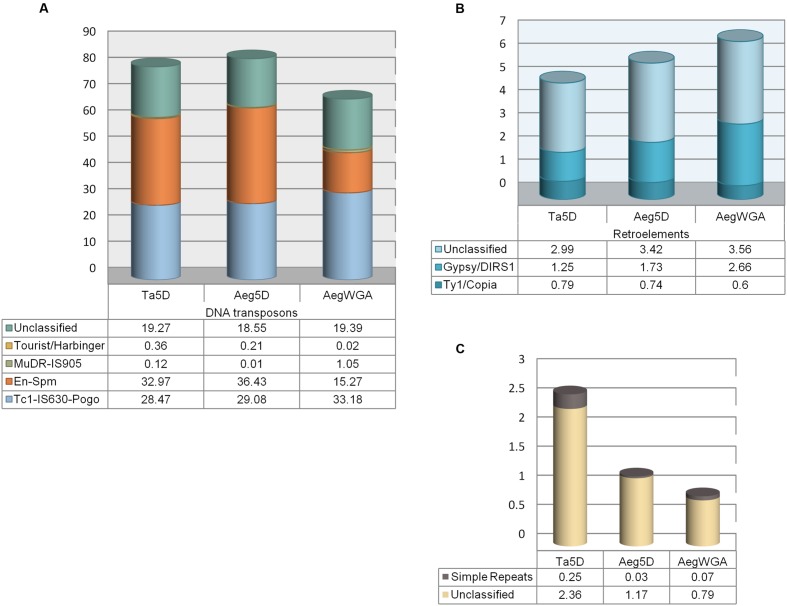
**Repetitive element distributions in miRNA stem–loops. (A)** Class II DNA transposons, **(B)** Class I retroelements, and **(C)** other repeat elements.

Repeat masking of the pre-miRNA sequences revealed that 91.38% of the total length of all putative stem–loops contained repetitive elements. Hairpins were particularly rich in Class II DNA transposons, accounting for 84.28% of the overall repeat content, while Class I LTR retrotransposons made up of only 5.89%. The most abundant Class II elements were En/Spm and TcMar, representing 36.43 and 29.08% of all stem–loop repeats, respectively; the Harbinger subfamily was also observed (0.21%, **Figure 1A**). Gypsy (1.73%) and Copia (0.74%) superfamilies, on the other hand, were the most prominent Class I retroelements (**Figure 1B**). Overall, of the 3,055 stem–loops representing 58 miRNA families, 2,913 (49 miRNA families) and 129 (15 miRNA families) were ‘repeat-related’ and ‘nonrepeat-related,’ respectively. In terms of miRNA families, 9 were categorized as ‘high confidence’ and 6 as ‘low confidence’ out of the total 58 families (**Table 1**).

**FIGURE 2:**
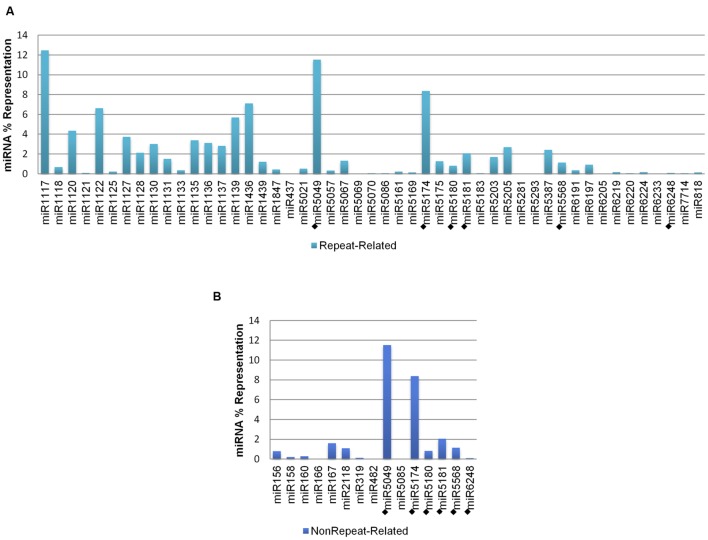
**Percent representations of *A. tauschii* 5D stem–loops. (A)** Repetitive, and **(B)** non-repetitive. Low confidence miRNAs are denoted by diamonds, ‘◆.’

Genomic representation (referred as ‘representation,’ hereafter) analysis was performed for all predicted *A. tauschii* 5D miRNA-coding regions (58 miRNAs; 3,055 stem–loops), including ‘repeat-related’ and ‘nonrepeat-related’ hairpins (**Table 1**). The representations of different miRNA families were observed to be variable, with 34 miRNAs contributing less than 1% to the overall representation. However, this value was as high as 12.47% for miR1117, which did not have any non-repetitive hairpins, but had the highest number of repetitive hairpins. In contrast, miR167, with the highest number of non-repetitive hairpins, did not have any repetitive hairpins and constituted only 1.60% of the overall representation. Thus, the observed variation of representations was largely due to the variation in repetitive stem–loops, as their contribution to the overall representation was much higher (95.81%). **Figure 2** demonstrates comparative miRNA representations separately for ‘repeat-related’ and ‘nonrepeat-related’ stem–loops. ‘Low confidence’ miRNA families are included in both graphs (denoted by diamonds, ‘◆’).

### Comparative Analysis of *Aegilops* and Bread Wheat 5D miRNAs

A major aim of this study was to analyze the conservation of miRNA coding regions across *A. tauschii* and *T. aestivum* 5D chromosomes. For this purpose, bread wheat 5D miRNAs were retrieved from a recent publication of our group (Kurtoglu et al., 2013). *In silico* miRNA prediction methodologies were the same for both *T. aestivum* and *A. tauschii* 5D miRNAs, enabling a comparison of the two datasets. The bread wheat 5D dataset consisted of a total of 60 miRNA families, with the corresponding 4,691 putative pre-miRNA coding regions, of which 3,692 were unique in sequence. Repeat analysis on this dataset revealed that 8 and 12 of the predicted miRNA families were ‘high confidence’ and ‘low confidence,’ respectively (**Table 1**).

The comparison of bread wheat 5D miRNAs (60) and *Aegilops* 5D miRNAs (58) revealed similar miRNA contents for both orthologous chromosomes. Additionally, for 48 miRNAs, at least one coding region was present on both *A. tauschii* and *T. aestivum* 5D chromosomes, suggesting a considerable level of conservation between bread wheat and its D genome progenitor. However, our observations also suggested that 10 miRNAs found on *A. tauschii* 5D chromosome may not be present on its *T. aestivum* ortholog; conversely, 12 miRNAs may be present on *T. aestivum* 5D but not on *A. tauschii* 5D (**Table 2**). While these differences may stem from chromosomal regions that were not covered by the survey sequences used for miRNA identification, it is also possible that one or more miRNA families may have been lost or emerged during the domestication and subsequent cultivation of the modern bread wheat. In terms of hairpins, putative miRNAs that were conserved in *A. tauschii* and *T. aestivum* 5D chromosomes also exhibited intriguing differences where 10 miRNA families were assigned to different categories (high confidence, low confidence and others with only repetitive hairpins) in two species. Of these, six miRNA families, namely miR1117, miR1130, miR1133, miR1139, miR5175, miR5205, were processed from exclusively repeat-related hairpins in *A. tauschii* but not in *T. aestivum*. In contrast, miR5180 and miR5181 families exhibited the opposite trend; in *T. aestivum* miR5180 and miR5181 families were generated exclusively by repeat-related hairpins. Two miRNA families, miR167 and miR2118 appeared to gain repetitive stem–loops in bread wheat. These observations suggest a dynamic nature of miRNA hairpins during wheat evolution.

Repetitive elements were observed to cover a slightly lower percentage of the cumulative length of all stem–loops in *T. aestivum* (88.83%), compared to its grass ancestor (91.38%). In bread wheat, similar to *A. tauschii*, Class II DNA transposons were the predominant repeat elements (81.19%), while LTRs constituted 5.03% of the repeats. Major Class I retroelement subclasses in hairpins were the same in both species, despite slight variations in overall distributions (1.25% for Gypsy; 0.79% for Copia in bread wheat, **Figure 1**). Likewise, *T. aestivum* and *A. tauschii* 5D pre-miRNAs harbored similar percentages of Harbinger and TcMar subclasses of DNA transposons, 0.36 and 28.47%, respectively in bread wheat. The overall percentage of the most prominent DNA transposon family, En/Spm, was slightly lower in bread wheat (32.97%). Interestingly, Mutator (MuDR) subclass of DNA transposons (0.01 vs. 0.12%) and simple repeats (0.03 vs. 0.25%) were almost 10-times as abundant in *T. aestivum* 5D pre-miRNAs as *A. tauschii* 5D pre-miRNAs (**Figures 1A,C**).

**FIGURE 3:**
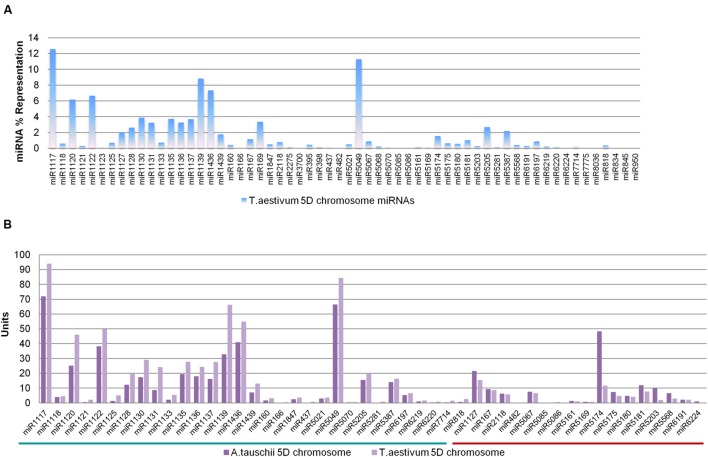
**Representations of wheat 5D stem–loops. (A)** Percent representations of bread wheat 5D miRNAs, and **(B)** Percent representations of miRNAs common to *A. tauschii* and *Triticum aestivum* 5D chromosomes. miRNAs with higher representations in *A. tauschii* or *T. aestivum* 5D chromosomes are emphasized with the green or red lines, respectively (Total miRNA repertoires of *Aegilops* and wheat 5D were accepted to be 577 and 748 units, respectively. miRNA representation values are expressed in units in the comparative bar graphs).

In order to compare the genomic representations of putative 5D miRNAs across bread wheat and *Aegilops*, representations of bread wheat miRNA families were also investigated. The 60 miRNA families putatively encoded by bread wheat 5D chromosome were represented by 4,691 pre-miRNAs, consisting of 309 ‘nonrepeat-related’ and 4,382 ‘repeat-related’ hairpins (**Table 1**). Similar to *A. tauschii* 5D miRNAs, miR1117 had a remarkable abundance among all miRNAs, accounting for 12.56% of all representation, while 40 bread wheat 5D miRNA families contributed less than 1% (**Figure 3A**). Among the miRNA families (48) commonly identified from both orthologous 5D chromosomes, representations were remarkably similar, as shown in **Figure 3**. Thirty-one miRNA families had higher representations in *T. aestivum* 5D chromosome compared to *A. tauschii* 5D chromosome, and vice versa for 17 miRNA families (**Figure 3B**).

### Comparative Analysis of *Aegilops* 5D miRNAs With Regard to the Entire Genome

To assess *A. tauschii* 5D chromosome miRNA content with regard to its whole genome, a total of 7,107,581 contigs from the recently published *A. tauschii* whole genome assembly (Jia et al., 2013) were used to predict miRNAs at the genome level. This resulted in the *in silico* identification of 80 miRNA families putatively encoded by a total of 4,868 pre-miRNAs, of which 4,068 were unique in sequence. Repeat element analysis suggested that 26 miRNA families were ‘high confidence’ and 8 were ‘low confidence’ (**Table 1**).

Putative *Aegilops* miRNAs identified in this study were then combined with previously reported *Aegilops* miRNAs (63 families in total; Dryanova et al., 2008; Kenan-Eichler et al., 2011; Jia et al., 2013) to define a comprehensive set of all currently known *Aegilops* miRNAs. This combined list contains 114 miRNA families, of which 51 are reported for the first time in this study (**Table 3**). Fifty-six families, out of 114, were not predicted from the 5D chromosome reads, suggesting that coding regions for these miRNAs may be located elsewhere in the *A. tauschii* genome. On the other hand, 5D chromosome putatively harbored pre-miRNA coding regions for more than half of the miRNA families (58 out of 114) reported for *A. tauschii* to date, which implies that *A. tauschii* 5D chromosome is relatively rich in pre-miRNA coding regions. Interestingly, seven miRNA families in the combined list were predicted exclusively from chromosome-specific 5D sequence reads, suggesting that in the absence of a finished quality genome sequence, survey sequences and sequence assemblies can complement and aid each other to provide a near-complete view of the genome. Additionally, these 5D miRNAs have not been reported in a previous small RNA sequencing study (Kenan-Eichler et al., 2011; Jia et al., 2013), which may indicate very low expression levels or highly tissue or environment-specific expression profiles, emphasizing the power of the genomic sequence-based prediction approaches in unlocking the complete miRNA contents of the genomes (**Supplementary Figure S1**).

**Table 3:** *Aegilops* whole genome miRNA data.

**(A) miRNAs newly identified from *Aegilops* WGA and 5D data**					
miR158	miR1122	miR437	miR5387	miR6248	miR5522
miR482	miR1125	miR5021	miR5568	miR7714	miR5523
miR5069	miR1131	miR5049	miR6191	miR818	miR5566
miR5161	miR1133	miR5085	miR6197	miR1039	miR6246
miR5183	miR1136	miR5086	miR6205	miR1123	miR7775
miR5293	miR1137	miR5174	miR6219	miR1138	miR845
miR1117	miR1139	miR5180	miR6220	miR165	
miR1118	miR1439	miR5205	miR6224	miR170	
miR1121	miR1847	miR5281	miR6233	miR415	
**(B) miRNAs identified in both previous studies and this study**					
miR1120	miR156	miR169	miR319	miR399	miR5169
miR1127	miR159	miR171	miR393	miR5057	miR5175
miR1128	miR160	miR172	miR394	miR5064	miR5181
miR1130	miR164	miR1878	miR395	miR5067	miR5200
miR1135	miR166	miR2118	miR396	miR5070	miR5203
miR1436	miR167	miR2275	miR398	miR5084	miR530

Curiously, the repeat content of the pre-miRNAs predicted from *Aegilops* whole genome assembly was much lower than that of pre-miRNAs predicted from the 5D chromosome alone (76.64 vs. 91.38%). This may suggest that pre-miRNAs putatively located on the 5D chromosome are rich in repetitive sequences. Compared to the whole genome, chromosome 5D related pre-miRNAs also exhibited considerable variation in the repeat subfamily distribution, where Class II DNA transposons were more abundant in contrast to Class I retroelements (84.28 vs. 68.91% and 5.89 vs. 6.87%, respectively). In particular, chromosome 5D appeared to accumulate more pre-miRNAs associated with En-Spm subfamily of DNA transposons, while overall, pre-miRNAs contained elements mostly from the TcMariner subfamily (33.18%) in the *A. tauschii* genome (**Figure 1**). In addition, LINE subclass of retroelements were only detected in assembly-predicted pre-miRNAs, despite in trace amounts (0.05%).

**FIGURE 4:**
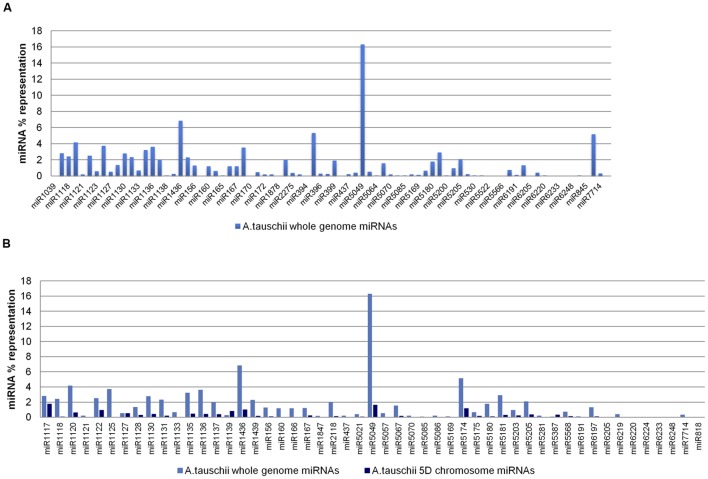
**Representations of *Aegilops* miRNAs. (A)** *Aegilops* miRNAs predicted from whole genome assembly, and **(B)** comparative representations of miRNAs in *Aegilops* 5D chromosome and whole genome (5D chromosome miRNA repertoire was accepted to constitute 14.32% of the overall *Aegilops* miRNA content. Comparative bar graphs show the percent representation on the 5D chromosome and *Aegilops* genome corresponding to each miRNA).

The 80 putative miRNA families, predicted from the *A. tauschii* whole genome assembly, were putatively processed from 993 ‘nonrepeat-related’ and 3,875 ‘repeat-related’ hairpins. Of the assembly-predicted miRNAs, miR5049 was found to be the most predominant, accounting for 16.31% of the overall representation, while 52 miRNA families contributed less than 1% each to the overall representation (**Figure 4A**). The miRNA families identified from both 5D chromosome reads and the whole genome assembly were further compared in terms of representation, except for four families (miR1127, miR1139, miR5387, miR6224) whose representations were unexpectedly lower at the whole genome level compared to the subgenomic level, likely resulting from sequencing-based overrepresentations in the input 5D reads, or from an overestimation in our analysis in relation to the contribution of a whole set of 5D miRNAs to the complete repertoire of *Aegilops* (set value: 14.32%). Two miRNA families, miR1117 and miR6248, had more than half of their coding regions on the 5D chromosome (**Figure 4B**).

### *In Silico* Expression and Target Analysis of Predicted miRNAs

In order to provide expressional evidence for putative miRNAs identified in this study, all unique *Aegilops* pre-miRNA sequences (6,569) of 87 miRNA families, collectively identified from whole genome and chromosome-specific predictions, were searched against two *Aegilops* expressed sequence databases: NCBI ESTs and whole transcriptome assembly (Jia et al., 2013). After stringent filtering (98% identity and 99% query coverage), for 16 miRNAs, at least one corresponding pre-miRNA gave a near-identical match to an expressed sequence, indicating the expression of these miRNAs (**Table 4**).

**Table 4:** Expressed sequence hit table of predicted *Aegilops* miRNAs.

miRNA names	EST
miR1117	gi|44888773|gb|AY534123.1|SEG_AY534122S2, gi| 442614136|gb|JX295577.1|, gi| 219814405| gb| FJ436986.1|
miR1118	gi| 442614136|gb|JX295577.1|
miR1120	gi|442614136|gb|JX295577.1|
miR1125	gi|442614136|gb|JX295577.1|
miR1128	Contig94874
miR1130	gi|300689672|gb|FJ898281.1|, gi|300689671|gb|FJ898280.1|, gi|300689650|gb|FJ898269.1|
miR1135	AEGTA02478, Contig23917
miR1136	gi|22038180|gb|AY013754.1|, Contig23917, AEGTA02478
miR1436	gi|442614136|gb|JX295577.1|
miR1439	gi|442614136|gb|JX295577.1|
miR437	gi|13447949|gb|AF338431.1|AF338431, gi|13447949|gb|AF338431.1|AF338431
miR5049	Contig115885, Contig29895
miR5064	AEGTA07380
miR5086	gi|21779916|gb|AF497474.1|
miR5174	Gb|JX295577.1, gb|GU211253.1
miR5180	Contig22176

*Hit names starting with “gi” were derived from NCBI A. tauschii (taxid:37682) EST database, while the others were derived from the transcriptome assembly of a recent study (Jia et al., 2013).*

The identification of transcripts targeted by miRNAs enables the elucidation of the biological roles of miRNAs in a functional context. Therefore, the transcripts potentially targeted by 51 miRNAs reported for the first time in this study were identified and annotated (Supplementary Table S3). Forty miRNAs, out of 46 to which at least one target was assigned, had multiple target transcripts, while miR1121, miR1123, miR5293, miR6233, miR6246, and miR818 targeted single transcripts. Functional annotation of the potential targets varied widely; however, majority of the targets were classified as transcription factors, ribosomal components and proteins involved in stress responses or plant metabolism (**Table 5**).

**Table 5:** List of targets regulated by multiple miRNAs.

miRNAs	Target accession	Target description	Target function
miR1117, miR1131	gb|EMT14610.1	Hexose carrier protein HEX6	Carbohydrate transmembrane transporter activity
miR1118, miR1439, miR5205, miR5568, miR6248	gb|EMT31416.1	Secologanin synthase	Monooxygenase, electron carrier, oxidoreductase, heme binding, metal ion binding (Fe) activity
miR1118, miR1439, miR5205	gb|EMT14706.1	Transcription factor IIIB 90 kDa subunit	DNA dependent transcriptinal regulation, TBP-class protein binding, zinc ion binding
miR1118, miR5085	gb|EMT12760.1	Sn1-specific diacylglycerol lipase alpha	Hydrolase activity, lipase activity
miR1122, miR5049, miR5205, miR5281, miR5568	gb|EMT23210.1	Obtusifoliol 14-alpha demethylase	Monooxygenase activity, iron ion binding, methyltransferase activity, electron carrier activity, heme binding
miR1125, miR5205	gb|EMT17935.1	rRNA biogenesis protein rrp5	RNA binding, mRNA processing
miR1133, miR6197	gb|EMT11624.1	Dynamin-related protein 3B	GTP binding, GTPase activity,
miR1137, miR1439, miR5205	gb|EMT26932.1	Putative serine/threonine-protein kinase	Serine/threonine kinase activity, kinase activity, ATP binding, transferase activity
miR1439, miR5049, miR5161, miR5174, miR5205, miR7714	gb|EMT12282.1	Putative E3 ubiquitin-protein ligase ARI8	Ligase activity, zinc ion binding, metal ion binding
miR1439, miR5049, miR5161, miR5205, miR5281, miR5568	gb|EMT13665.1	Pleiotropic drug resistance protein 3	ATP binding, ATPase activity
miR1439, miR6248	gb|EMT28996.1	Two-component response regulator ARR2	Regulation of seed germination, chromatin binding, DNA binding, regulation of transcription, DNA-dependent, phosphorelay signal transduction
miR1439, miR5161	gb|EMT07281.1	Putative laccase-9	Hydroquinone:oxygen oxidoreductase activity, metal ion (Cu) binding
miR1439, miR6248	gb|EMT02609.1	Monosaccharide-sensing protein 3	Transmembrane transporter activity
miR1439, miR5049, miR5161, miR5174, miR7714	gb|EMT13168.1	Hexokinase-8	Carbohydrate metabolic process, ATP binding, kinase activity, transferase activity
miR1439, miR5049, miR5205	gb|EMT27710.1	Cyclin-L1-1	Response to salt stress, stomatal lineage progression, post-translational protein modification, photoperiodism, flowering, regulation of cell cycle, regulation of transcription, catalytic activity, cyclin-dependent protein serine/threonine kinase regulator activity
miR1439, miR5049, miR6248	gb|EMT09857.1	Heat shock 70 kDa protein 4L	Response to stress, ATP binding, nucleotide binding
miR1439, miR5049, miR5205, miR6248	gb|EMT13688.1	Putative receptor-like protein kinase	ATP binding, nucleotide binding, polysaccharide binding, protein kinase activity, transferase activity
miR5049, miR6248	gb|EMT00359.1	E3 ubiquitin-protein ligase	Ligase activity, zinc ion binding, metal ion binding
miR5049, miR5205	gb|EMT23266.1	Eukaryotic translation initiation factor 2 subunit alpha	RNA binding, translation initiation factor activity,
miR5049, miR5205, miR5568, miR6220	gb|EMT32034.1	Vacuolar sorting-associated protein 11-like protein	Response to salt stress, vegetative to reproductive phase transition of meristem, vesicle-mediated transport, vacuole organization, golgi organization, transporter activity, zinc ion binding, metal ion binding, catalytic activity
miR5049, miR5205, miR6248	gb|EMT11495.1	ATP-dependent DNA helicase 2 subunit 1	Response to heat, telomere maintenance, DNA repair, helicase activity, DNA binding, hyrolase actvity
miR5049, miR5161, miR5174, miR7714	gb|EMT28147.1	GDSL esterase/lipase	Hydrolase activity, lipase activity
miR5161, miR5180	gb|EMT27792.1	Putative RNA-dependent RNA polymerase 1	RNA-directed RNA polymerase activity
miR5205, miR5281	gb|EMT14098.1	U-box domain-containing protein 12	Transferase activity, protein kinase activity, ubiquitin-protein ligase activity, ATP binding
miR5568, miR6220	gb|EMT28796.1	Ferredoxin-dependent glutamate synthase, chloroplastic	Oxidoreductase activity, catalytic activity, glutamate biosynthetic process
miR5568, miR6197, miR6224	gb|EMT05838.1	Mitochondrial Rho GTPase 1	GTPase activity, GTP binding, calcium ion binding, hydrolase activity
miR6220, miR6224	gb|EMT01896.1	Cysteine-rich receptor-like protein kinase 36	Serine/threonine kinase activity, kinase activity, ATP binding, transferase activity

### Expression Patterns of Selected miRNAs in Response to Drought

**FIGURE 5:**
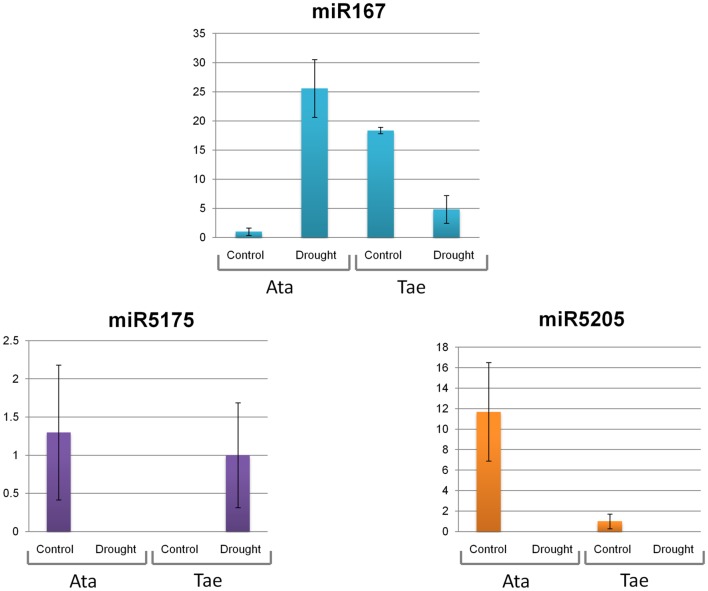
**Expression levels of miR167, miR5175, and miR5205 in *A. tauschii* and *T. aestivum* seedlings in response to drought stress.** Ata: *A. tauschii*, Tae: *T. aestivum* var. CS.

**FIGURE 6:**
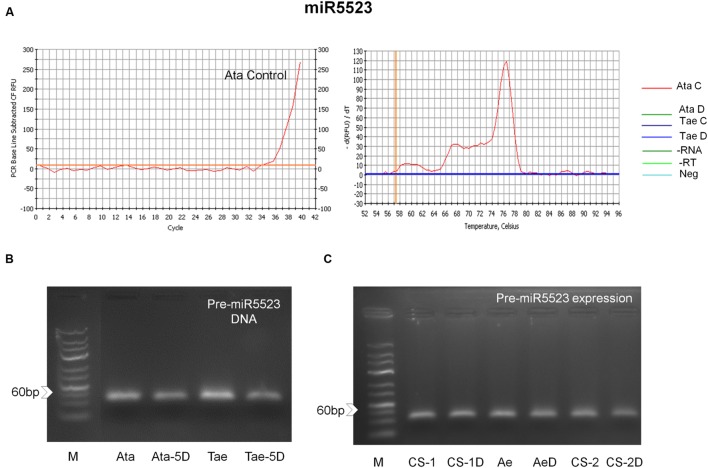
**miR5523 expression and coding regions in *A. tauschii* and *T. aestivum* var.** CS **(A)** real time amplification curve showing mature miR5523 expression in *A. tauschii*, and **(B)** pre-miR5523 PCR amplicons showing that 5D chromosome-located miR5523 coding regions are present in both species. **(C)** Pre-miR5523 expression in both species in control and 4 h shock drought stress conditions (CS-1: Chinese Spring 1 leaf stage control; CS-1D: Chinese Spring 1 leaf stage drought; Ae: *A. tauschii* 1 leaf stage control; AeD: *A. tauschii* 1 leaf stage drought; CS-2: Chinese Spring 2 leaf stage control; CS-2D: Chinese Spring 2 leaf stage drought; Neg: Negative control; -RT: No RT control; -RNA: No RNA control).

The expression patterns of four putative miRNAs, miR167, miR5175, miR5205 and miR5523, were investigated via qRT-PCR in response to 4-h shock drought application in whole seedlings of *A. tauschii* and *T. aestivum*. The expressions of miR167, miR5175, and miR5205 were observed in both species, while miR5523 was expressed only in *A. tauschii*. At this point, it is important to note that the expressions of these miRNAs could be anywhere from the genome, not restricted to 5D chromosomes. All four miRNAs were drought-responsive (**Figures 5** and **6A**). Under normal conditions, miR167 expression was 18 fold higher in *T. aestivum* than *A. tauschii*. Upon drought, however, miR167 was downregulated in *T. aestivum* (fourfold) but upregulated in *A. tauschii* (26 fold), likely resulting in a significant difference in miR167 accumulation in these two species. miR5175 exhibited an opposite trend in expression; the expression of this miRNA was detectable only under normal conditions in *A. tauschii* and only under drought stress in *T. aestivum*. In both species, miR5205 was downregulated in response to drought, where the downregulation was much more pronounced in *A. tauschii*. Similar to miR5175, miR5523 expression, detected only in *A. tauschii*, was either completely lost or reduced to trace amounts under drought stress.

The expression of miR5523 was not observed in *T. aestivum* seedlings under control or drought stress conditions. However, we cannot exclude the possibility that miR5523 is expressed under highly tissue-, condition- or developmental stage-specific circumstances in bread wheat. Indeed, pre-miR5523 coding regions were observed for both species, some of which were also located on the orthologous 5D chromosomes (**Figure 6B**). Pre-miR5523 expression was also evident in both species at multiple growth stages under control and drought conditions (**Figure 6C**). These observations may indicate that the expression of miR5523 might have been lost in modern bread wheat due to an interference with the downstream processing of its pre-miRNA under control conditions. Overall, this study provides the first report of expression of miR5523 and miR5175 in *A. tauschii* and *T. aestivum*, respectively, and the first experimental verification of miR5205 in both species.

## Discussion

*Aegilops tauschii*, also known as Tausch’s goatgrass, is a wild, diploid grass. Around 8,000 years ago, wild *A. tauschii* populations spontaneously hybridized with the allotetraploid emmer wheat *T. turgidum*, forming one of the pioneering food crops of today, the hexaploid bread wheat *T. aestivum* (Jia et al., 2013; Marcussen et al., 2014). Bread wheat D genome is therefore highly similar to its progenitor, *A. tauschii*, making this wild species of substantial interest to wheat researchers. Unlike the D genome of bread wheat, being the least polymorphic of the three subgenomes, *A. tauschii* populations exhibit remarkable genetic variation, thereby representing a rich source of alleles for wheat improvement (Dvorak et al., 1998; Akpinar et al., 2014).

Post-transcriptional regulation of gene expression is a fundamental molecular process for the proper functioning of organisms. Small, non-coding RNAs called the microRNAs, or miRNAs, are central to these regulatory circuits, playing important roles in various physiological processes, including drought response (Yao et al., 2007; Wilusz et al., 2009; Ding et al., 2013; Rogers and Chen, 2013). Therefore, identification and characterization of miRNAs in many species have been a hotspot of research in the last decade (Kawaji and Hayashizaki, 2008; Yao and Sun, 2012). Several groups have focused on the identification of bread wheat miRNAs, revealing a total of 213 families, of which 158 were experimentally verified (Yao et al., 2007; Dryanova et al., 2008; Wei et al., 2009; Xin et al., 2010; Yin and Shen, 2010; Kenan-Eichler et al., 2011; Schreiber et al., 2011; Vitulo et al., 2011; Gupta et al., 2012; Kantar et al., 2012; Lucas and Budak, 2012; Tang et al., 2012; Kurtoglu et al., 2013; Li et al., 2013; Pandey et al., 2013). Still, *Aegilops* miRNA pool has just begun to unlock. Although *Aegilops* miRNAs can deliver important clues into wheat genome function and evolution and can potentially be targeted for crop improvement, only a small number of *Aegilops* miRNAs have been reported to date (Dryanova et al., 2008; Kenan-Eichler et al., 2011; Jia et al., 2013). Here, we identified *A. tauschii* miRNAs at the genomic and subgenomic levels, using a homology-based *in silico* strategy. We also compared the putative miRNA content of the *A. tauschii* 5D chromosome to its modern ortholog, *T. aestivum* 5D chromosome to gain insight into the miRNA evolution of wheat genomes. Four selected miRNAs were further verified by experimental approaches and their expression changes in response to drought have been shown in *T. aestivum* and *A. tauschii* seedlings.

In our study, a cumulative number of 87 *Aegilops* miRNA families were computationally identified, of which 51 had not been previously reported (**Table 3**), considerably expanding our knowledge on *Aegilops* miRNAs. Notably, over half of the miRNA families that were previously reported (36 out of 63) were also identified in this study, supporting the reliability of our *in silico* identification strategy (**Supplementary Figure S1**). Putative miRNAs identified from the 5D chromosome of *A. tauschii* (58 families) comprised more than half of all reported *A. tauschii* miRNAs so far (114 families in total), which marks 5D as a potentially pre-miRNA rich chromosome.

Of the 58 and 60 miRNA families identified from *A. tauschii* and *T. aestivum* 5D chromosomes, 48 families were commonly identified from both chromosomes, pointing out to the close evolutionary relationship between these related D genomes. Ten miRNA families found in *A. tauschii* 5D chromosome but not in *T. aestivum* 5D, and 12 more families vice versa, entail further research, as some miRNAs within these families may have been lost, gained or translocated to non-syntenic regions through wheat miRNA evolution. It is important to note that the NGS reads used to predict these putative miRNAs may not necessarily cover the entirety of chromosomes, and a potential translocation event could still be missed even in the presence of finished quality chromosome sequences. Therefore, experimental validation of these families should provide a clearer picture, which may reveal evolutionary footprints, suggesting a mechanism for miRNA origin, before the reference genome sequences of these organisms are released (**Table 2**).

*Triticeae* are notable for their highly repetitive genomes, comprised of typically >80% repeat elments (Mayer et al., 2014). Repetitive elements have also been suggested to promote the formation of new genes or pseudogenes, contributing to genome evolution (Wicker et al., 2011). Recent findings suggests that miRNA gene evolution may also be driven by the activities of transposons (Li et al., 2011). Therefore, in this study, we investigated the repeat content of the predicted pre-miRNAs, which revealed the presence of large quantities of Class II DNA transposons, consistent with previous observations (Vitulo et al., 2011; Kantar et al., 2012; Lucas and Budak, 2012; Kurtoglu et al., 2013, 2014). In general, Class I retrotransposons are prevalent in plant genomes. The association of Class II elements in miRNA coding regions is, hence, noteworthy, indicating that Class II elements may indeed contribute to miRNA evolution (Li et al., 2011). Repetitive elements constitute a slightly higher portion of the *A. tauschii* 5D stem–loops (91.38%), than that of bread wheat (88.83%), in line with previous observations on the overall repeat content of the wheat genomes (Jia et al., 2013; Luo et al., 2013; Mayer et al., 2014). Copy numbers of most repeat families are suggested to be dynamic, exhibiting differential proliferation in A, B and D genomes through wheat evolution (Li et al., 2004; Charles et al., 2008). In our study, both Class I and Class II elements were slightly more abundant in putative pre-miRNAs identified from *A. tauschii* 5D chromosome, compared to its bread wheat ortholog. However, MuDR subclass of DNA transposons, simple repeats and other unclassified repetitive elements were more abundant in *T. aestivum* 5D pre-miRNAs, which may indicate that Transposable Element (TE)-driven proliferation of stem–loops containing these repeats might have occurred in bread wheat D genome following polyploidization (**Figure 1**). Compared to the whole genome assembly-derived *A. tauschii* pre-miRNAs, chromosome 5D appeared to be richer in pre-miRNAs containing repeat elements. Interestingly, 5D chromosome pre-miRNAs contained mostly En/Spm subfamily of repeats, whereas at the genome level, putative pre-miRNAs were mostly associated with TcMar type repeat elements in *A. tauschii* (**Figure 1A**). Conversely, LTR elements, in particular Gypsy subfamily, were scarcer in chromosome 5D pre-miRNAs, in comparison to the putative pre-miRNAs encoded by the whole genome.

Putative 5D miRNA families of *A. tauschii* (58) and *T. aestivum* (60) revealed a marked abundance of miR1117 family among the representations of all miRNA families. Additionally, miRNA families commonly identified from both orthologous chromosomes exhibited similar representations in general, although 17 miRNAs were more abundant in *A. tauschii* 5D, while 31 were represented more on the 5D chromosome of bread wheat (**Figure 3B**). The differential proliferation of stem–loops for certain families may be linked to TE-activity, in particular TE-expansion following polyploidization (Li et al., 2004). Curiously, of the 51 miRNA families common to *A. tauschii* whole genome assembly (80 families) and 5D sequence read (58 families) predictions, two miRNAs (miR1117 and miR6248) were highlighted for having more than 50% of their coding regions on the 5D chromosome. Overall, repeat analysis and miRNA representations (91.38% in *A. tauschii* 5D, 88.83% in *T. aestivum* 5D, 76.64% in *A. tauschii* whole genome) suggest *A. tauschii* 5D as a repetitive hairpin rich chromosome of the genome, harboring more ‘repeat-related’ stem–loops in comparison to its bread wheat ortholog. These observations are consistent with the previous reports on genome wide repeat contents of these species (Li et al., 2004; Choulet et al., 2010; Brenchley et al., 2012; Jia et al., 2013; Luo et al., 2013).

A drawback of our miRNA prediction method from genomic sequences is that predictions can include miRNA-like sequences that are silent due to the lack of intact promoters. In order to provide expressional evidence for the putative *A. tauschii* miRNAs identified in this study, respective pre-miRNA sequences were compared to *Aegilops* ESTs and transcriptome assembly (Jia et al., 2013). Under stringent criteria (98% identity and 99% query coverage), 16 miRNAs (out of 87) were found to give almost exact matches to these expressed sequences. The expressions of six of these miRNAs (miR1120, miR1128, miR1130, miR1135, miR1436, and miR5064) have also been shown previously in *Aegilops* small RNA libraries (Jia et al., 2013). Out of 51 miRNA families reported for the first time in this study, *in silico* evidence was provided for 10 miRNA families. We cannot exclude the possibility that the remaining miRNA families are indeed expressed but the expression is highly tissue, developmental stage and/or environment specific (**Table 4**, **Supplementary Figure S1**).

MicroRNAs take part in various physiological processes through regulation of their targets. Thus, identification of target transcripts is crucial to elucidate specific functions of respective miRNAs. Forty-six of 51 newly identified *A. tauschii* miRNAs were assigned putative targets (Supplementary Table S3). For 40 miRNAs, multiple target transcripts were predicted, suggesting multiple regulatory functions. On the other hand, 27 of the transcripts were targeted by more than one miRNA, which may indicate crosstalk between miRNA regulatory networks (**Table 5**). Functional annotation of these target transcripts revealed various molecular functions, including transporter activity (miR1131), protein kinase activity (miR1137/miR1439/miR5205), ligase activity (miR5180), hydrolase activity (miR5568/miR6197/miR6224), oxidoreductase activity (miR1118/miR1439/miR5205), RNA binding (miR1125/miR5205) and drug resistance (miR482/miR5049). Several others indicated roles in response to stress conditions, such as salt (miR5049/miR5205/ miR5568/miR6220; gb|EMT32034.1) and heat (miR5049/miR5205/miR6248; gb|EMT11495.1). Additionally, Aquaporin (gb|EMT21244.1), a drought related protein (Kantar et al., 2011a), was targeted by miR6197 (**Table 5**, Supplementary Table S3).

Four miRNAs (miR167, miR5175, miR5205, miR5523) were selected for quantification of expression in response to drought stress, the most prevalent stress condition causing severe yield losses worldwide. Uncovering novel dehydration-responsive molecular mechanisms in different species holds great significance and can contribute to crop improvement (Kantar et al., 2011a; Budak et al., 2015b). To date, the role of plant miRNAs in drought has been highlighted in various studies (Budak and Akpinar, 2011) and several dehydration-related miRNAs were identified in a wild relative of bread wheat, as well as two closely related species (*T. dicoccoides, Hordeum vulgare, Brachypodium distachyon*; Unver and Budak, 2009; Kantar et al., 2010, 2011b). Of the four selected miRNAs, miR167 is conserved among plants, including *A. tauschii* and wheat, and has been implicated in abiotic and biotic stress responses (Yao et al., 2007; Wei et al., 2009; Xin et al., 2010; Kenan-Eichler et al., 2011; Gupta et al., 2012; Tang et al., 2012; Jia et al., 2013; Li et al., 2013; Khaksefidi et al., 2015). While the involvement of miR167 in drought response has been reported in *Arabidopsis*, but not in wheat (Liu et al., 2008; Kinoshita et al., 2012), miR5175, miR5205, and miR5523 have not been characterized at all. Under control conditions, miR167 expression appeared to be relatively high in *T. aestivum*, similar to previous observations that syntetic hexaploids (*T. turgidum durum* ×*A. tauschii)* had higher miR167 levels, compared to the diploids (Kenan-Eichler et al., 2011). Upon drought, miR167 was downregulated in *T. aestivum*, however, its expression was remarkably stimulated in *A. tauschii* (**Figure 5**). Conversely, the expression of miR5175 was downregulated in *A. tauschii* but upregulated in *T. aestivum* in response to drought (**Figure 5**). These two miRNAs can point out to ancient regulatory pathways in the D genome progenitor that might have been modulated in the modern bread wheat. To date, miR5175 has been reported only in *A. tauschii* and a closely related model grass species, *B. distachyon* (Baev et al., 2011; Jia et al., 2013). It is tempting to speculate that further characterization of miR5175 may reveal regulatory circuits specific to wheat and its close relatives. On the other hand, miR5205 has been reported only in *Medicago truncatula*, but was also suggested to be conserved in other plants, as well, such as *Zea mays* (Devers et al., 2011). miR5205 was downregulated in both *T. aestivum* and *A. tauschii* under drought stress conditions, providing the first experimental evidence for its expression in wheat species (**Figure 5**). The shared patterns of expression point out to a conserved regulation mechanism in bread wheat and its ancestor that can help elucidate the complex drought response of wheat through further characterization. miR5175 had been reported by Jia et al. (2013) in *A. tauschii*; however, its expression in wheat had not been previously shown until now. Due to its drought specific expression in bread wheat, miR5175 might have eluded identification from previous small RNA sequencing studies, which demonstrates the utility of genomic sequences in miRNA prediction and identification.

The expression of miR5523, previously identified in *Oryza sativa* (Wei et al., 2011), could only be verified in *A. tauschii* under normal conditions (**Figure 6A**). miR5523 was totally suppressed when plants were exposed to drought, indicating a negative regulatory role in the drought response. While the expression of this miRNA was not detected in control or drought-stressed *T. aestivum* seedlings, pre-miRNA coding region was conserved in the bread wheat genome (**Figure 6B**). Furthermore, pre-miR5523 expression was observed in both *T. aestivum* and *A. tauschii* both under control and stress conditions, suggesting that pre-miR5523 can nonetheless be processed into mature miR5523 under specific conditions in bread wheat. During normal growth, however, the expression of mature miR5523 might have been blocked likely through an interference with the downstream pre-miRNA processing in bread wheat. Whether this interference is an intentional level of self-regulation or is caused by disruptions within the processing machinery remains elusive at this time.

## Author Contributions

HB conceived and designed the experiment, drafted manuscript and is involved in analysis. BA performed the analysis and drafted manuscript.

## Conflict of Interest Statement

The authors declare that the research was conducted in the absence of any commercial or financial relationships that could be construed as a potential conflict of interest.

## Footnotes

1 www.repeatmasker.org

2 www.phrap.org/phredphrapconsed.html

3 ftp://ftpmips.helmholtz-muenchen.de/plants/REdat/

4 http://plantgrn.noble.org/psRNATarget/

5 http://www.ebi.ac.uk/QuickGO/

## Supplementary material

FIGURE S1 **Venn diagram depicting comparison of different miRNA datasets. (A)** Predictions from *A. tauschii* whole genome assembly. **(B)** Predictions from *A. tauschii* 5D reads. **(C)** *Aegilops* miRNAs reported in previous studies.

## Abbreviations

CS: Chinese spring
En/Spm: enhancer/suppressor-mutator
EST: expressed sequence tag
LTR: long terminal repeat
MFE: minimum free energy
miRNA: microRNA
MuDR: mutator
NGS: next generation sequencing
pre-miRNA: precursor miRNA
qRT-PCR: quantitative real time reverse transcription PCR
TcMar: TcMariner
