# Supplementary figures and images for: Dissecting miRNAs in Wheat D Genome Progenitor, Aegilops tauschii
Source: Front Plant Sci. 2016 May 4;7:606. doi: 10.3389/fpls.2016.00606 (PMC4855405; doi:10.3389/fpls.2016.00606)

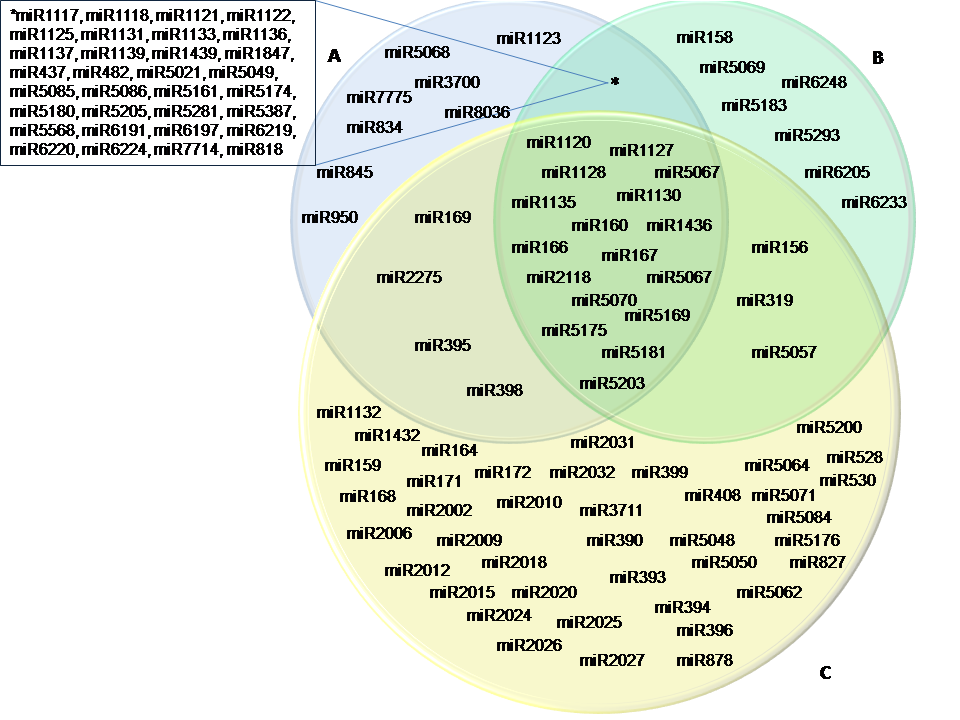

Supplement: FIGURE S1 — Venn diagram depicting comparison of different miRNA datasets. (A) Predictions from A. tauschii whole genome assembly. (B) Predictions from A. tauschii 5D reads. (C) Aegilops miRNAs reported in previous studies. [file Image_1.TIF]
